# The breastfeeding experience of women with multiple pregnancies: a meta-synthesis of qualitative studies

**DOI:** 10.1186/s12884-024-06697-4

**Published:** 2024-07-22

**Authors:** Ruxue Bai, Yifan Cheng, Siyu Shan, Xinmiao Zhao, Jun Wei, Chunling Xia

**Affiliations:** Department of Obstetrics and Gynecology, Shengjing Hospital of China Medical University, Shenyang City, 110004 Liaoning Province China

**Keywords:** Breastfeeding, Multiple birth, Twins, Qualitative research, Systematic review, Meta-synthesis

## Abstract

**Background:**

The experiences and challenges associated with breastfeeding multiple births can be considerably more complex than those of singletons. Multiple births refer to the delivery of more than one offspring in a single birth event. Emphasizing the needs and experiences of mothers with multiple births during breastfeeding can enable healthcare providers to design targeted interventions that enhance breastfeeding rates. However, existing breastfeeding and health education resources and practices do not fully meet the needs of women who breastfeed multiples. This review aimed to review and synthesize qualitative studies on the breastfeeding experiences of women with multiple births.

**Methods:**

A systematic search was conducted in 10 electronic databases for papers published from the inception of the database to March 2024. The Joanna Briggs Institute Critical Appraisal Checklist for Qualitative Research was utilized to evaluate the methodological quality of the studies included. The thematic synthesis method of Thomas and Harden was employed to integrate and analyze the included literature to derive new categories and conclusions.

**Findings:**

Eight studies met the inclusion criteria and quality assessment criteria for this study. Through the integration of their results, four themes were identified: the choice and willingness to breastfeed multiple births; the challenges of breastfeeding multiple births; stage management and individualised adaptation of breastfeeding; and the experience of support.

**Conclusion:**

Throughout the feeding process from pregnancy to the postpartum period, mothers with multiple births often have predominantly negative experiences with breastfeeding. Consequently, hospitals should create a multidisciplinary follow-up team comprising obstetrics, neonatology, psychology, and community services to offer specialized and personalized support to these women at various stages.

**Systematic review registration:**

[https://www.crd.york.ac.uk/PROSPERO/], identifier [PROSPERO 2024 CRD42024520348].

**Supplementary Information:**

The online version contains supplementary material available at 10.1186/s12884-024-06697-4.

## Introduction

Over the past three decades, the global incidence of multiple pregnancies has significantly increased due to delayed childbearing, advancements in assisted reproductive technologies, and the use of ovulation-inducing drugs [1]. In Italy, the rate of twin pregnancy increased from 1.28% in 2015 to 2.48% in 2020 [2], and in the United States, it surged by 70% from 1980 to 2019 [3, 4]. Similarly, in China, the incidence of multiple pregnancies grew by 60% between 2009 and 2019 [1]. Multiple pregnancies are categorized as high risk, with 60% of twins being born prematurely before 37 weeks and 75% of triplet pregnancies born before 35 weeks [5], significantly increasing the risk of adverse neonatal outcomes.

Exclusive breastfeeding is deemed the optimal feeding method for newborns, particularly beneficial for the development of premature infants [6]. The American Academy of Pediatrics strongly recommends exclusive breastfeeding for the first six months, highlighting its effectiveness in reducing the risk of various health issues in premature infants, including sepsis, necrotizing enterocolitis, severe retinopathy of prematurity, metabolic syndrome, long-term growth retardation, and neurodevelopmental disorders [7]. However, international studies from countries such as Japan [8, 9], Italy [2], Indonesia [10], South Korea [11], Sweden [12], China [13], and those with high breastfeeding rates such as Ghana [14, 15] revealed that exclusive breastfeeding rates among women with multiple births consistently lag behind those of women with single births at all measured intervals. Specifically, exclusive breastfeeding rates at six months were less than 17%, significantly below the World Health Organization’s target of 50% [16]. Therefore, enhancing the breastfeeding rates among women with multiple births is crucial.

Research indicates that the low breastfeeding rates among women with multiple births are attributable to factors related to mothers, newborns, and support systems. Breastfeeding self-efficacy is a critical determinant of exclusive breastfeeding rates in this group [10, 17]. High self-efficacy signifies that mothers possess confidence and proficiency in breastfeeding multiple infants and are proactive in addressing challenges during the feeding process. The perception of insufficient milk supply is the predominant reason for cessation of breastfeeding among these women, with many doubting their ability to produce adequate milk for multiple infants [2, 15, 18]. In contrast, studies have shown that oxytocin levels in mothers of twins are double those in mothers of singletons, leading to doubled milk production. By six months postpartum, these mothers can produce between one to two kilograms of milk daily, sufficient for the needs of two infants [19]. The primary complication associated with multiple births is preterm delivery, which often results in the separation of mother and infant. This separation complicates the initiation of critical early breastfeeding practices, including immediate sucking, skin-to-skin contact, and early milk production. Furthermore, the persistent immature feeding behaviors of preterm infants—characterized by weak sucking, lethargy, and poor tolerance—continue to challenge breastfeeding efforts post-discharge, contributing to a lower rate of exclusive breastfeeding among mothers of multiples [20]. Comprehensive medical [21], family [2], and social support [15] are essential to enhance breastfeeding rates among women with multiple births.

As the number of infants increases, the complexities and challenges of infant care, particularly breastfeeding, intensify with multiple births. It requires greater physical strength, belief in breastfeeding and support systems from the mother [2]. After encountering numerous challenges, mothers are more likely to cease breastfeeding [20]. A deep understanding of the breastfeeding experiences and needs of women with multiple births can enable healthcare professionals and breastfeeding specialists to identify issues early and offer personalized support services, thereby enhancing breastfeeding rates and duration [22]. There are already qualitative studies on the real experiences of breastfeeding among women with multiple births, but a single qualitative study cannot fully reflect all experiences of this group. There is still a lack of integration of relevant research results. This study adopts a meta-synthesis approach to consolidate qualitative research in this area, aiming to provide a more comprehensive and profound insight into the real experiences of breastfeeding among women with multiple births. This approach serves as the foundation for developing targeted interventions, relevant social support, and psychological care in the future.

## Methods

This systematic review was registered prospectively with the International Prospective Register of Systematic Reviews (PROSPERO) with registration number CRD42024520348. The Enhancing Transparency in Reporting the Synthesis of Qualitative Research (ENTREQ) checklist (Supplementary Table 1) was used to report the process and results of synthesis and enhance transparency [23].

### Search strategy

We conducted comprehensive searches across ten widely-used Chinese and English databases, including the China Biomedical Database (CBM), China National Knowledge Infrastructure (CNKI), Wanfang Database, VIP Database, PubMed, Web of Science, Embase, Cumulative Index to Nursing and Allied Health Literature (CINAHL), the Joanna Briggs Institute (JBI) Evidence-Based Healthcare Center Database, and PsycINFO, to collate qualitative studies on breastfeeding among mothers with multiple births. The search covered the period from the inception of each database to March 2024. We employed both MeSH descriptors and free-text terms, and systematically reviewed the reference lists of the included studies to ensure a thorough literature search. The search terms included: “pregnancy” “twins” “multiple births” “higher order pregnanc*” “breastfeeding” “breastfeed*” “breast-feed*” “experience” “need*” “feeling” “qualitative research” “qualitative study” “ground theory” “interview*” “focus groups” “phenomenon” “ethnography”. The search strategy for PubMed is detailed in Table 1.

**Table 1:** Search strategy in PubMed

	search terms
#1	“Twins”[Mesh] OR Pregnant Women[Mesh Terms] OR multiple births[Title/Abstract] OR higher order pregnanc*[Title/Abstract]
#2	“Breast Feeding”[Mesh] OR breast feed[Title/Abstract] OR breastfeeding[Title/Abstract]
#3	experience[Title/Abstract] OR feeling[Title/Abstract] OR need*[Title/Abstract] OR qualitative research[Title/Abstract] OR qualitative study[Title/Abstract] OR ground theory[Title/Abstract] OR interview*[Title/Abstract] OR focus groups[Title/Abstract] OR phenomenon[Title/Abstract] OR ethnography[Title/Abstract]
#4	#1 AND #2 AND #3

### Inclusion and exclusion criteria

We applied the PICOS framework, as recommended by the Joanna Briggs Institute (JBI) Evidence-Based Healthcare Center [24], to establish the inclusion and exclusion criteria for the studies. The inclusion criteria were as follows: (1) Population: women with multiple gestations, including twins, triplets, and higher-order multiples; (2) Interest of phenomena: the experiences, feelings, and needs of these women concerning breastfeeding multiple infants; (3) Context: the specific settings such as hospital wards, outpatient departments, or the homes of the pregnant women; (4) Study design: qualitative research, including various qualitative research literature using descriptive analysis, phenomenology, grounded theory, ethnography, the qualitative part of mixed research and so on. The exclusion criteria were as follows: (1) literature not published in Chinese or English; (2) literature for which the full text is not available; (3) literature that has been published multiple times. (4) the literature quality evaluation grade is C.

### Literature selection and data extraction

All search records were imported into the reference management software NoteExpress, and duplicate records were removed by the principal investigator (BRX). Two researchers (BRX and SSY), who had undergone systematic training in evidence-based practice methods, independently conducted literature searches using the specified terms. They then rigorously applied the inclusion and exclusion criteria to screen the literature. This involved a meticulous review of the titles and abstracts for preliminary screening to discard irrelevant studies. Subsequently, we conducted a full-text review to finalize the selection of included studies. Any disagreements were resolved through discussion or by consulting a third researcher (CYF).

The data extraction process included the first author’s name, publication date, country, qualitative research method, research subject, phenomenon of interest, and key findings.

### Quality appraisal

Two researchers (BRX and SSY) worked independently using the Joanna Briggs Critical Assessment tool for Methodological Quality Assessment [24], which is widely applicable to the evaluation of qualitative research. The evaluation content consisted of 10 items, each of which is evaluated as “yes”, “no”, “unclear”, or “not applicable”. The quality of the literature is divided into A, B, and C levels. If all the standards are fully met, the possibility of bias is the smallest, and the quality level is “A”. If some of the above standards are met, the possibility of bias is moderate, and the quality level is “B”. If none of the above standards are met, the possibility of bias is the greatest, and the quality level is “C”. In the event of any disagreements, a third researcher (CYF) will be responsible for making a joint decision.

### Translation

During the data analysis process, two researchers translated the results and discussion sections of the Chinese literature into English, compared their translations, and negotiated the most appropriate version. After that, the two researchers then translated the English back into Chinese, compared the back-translated content with the original Chinese text, and discussed and resolved any disagreements. Based on the discussion results, the English translation was revised. Where cultural concepts could not be translated, native English speakers were consulted for translation advice, but this was not encountered during the translation of this paper. All researchers were staff in the field of obstetrics and were proficient in English.

### Data synthesis

The data was synthesized using Thomas and Harden’s thematic synthesis methods [25]. A thematic synthesis was used to interpret multiple findings and develop analytical themes enhancing understanding [26]. the development of analytical themes that enhance understanding. Initially, two researchers (BRX and SSY) imported all relevant content from the “Results” and “Conclusions” sections of the included studies into NVivo11. They then coded the findings line by line based on their meaning and content. These codes were systematically analyzed and organized to identify similarities and differences, which were grouped into related areas to form descriptive themes. Analytical themes were subsequently developed inductively by synthesizing the study findings and elucidating their meanings. Discrepancies were deliberated upon by two researchers and, where necessary, a third researcher (ZXM) was consulted to resolve any contradictions.

## Findings

### Literature search

**Fig. 1:**
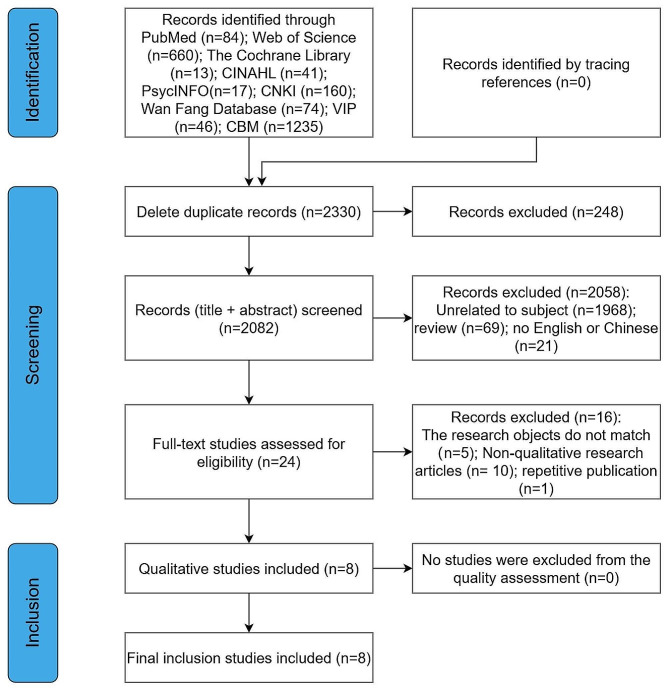
Flow diagram of the search strategy and results

We first obtained a preliminary total of 2,330 studies through database search and reference literature review. Then, using NoteExpress software, 248 duplicate studies were excluded. By reviewing the titles and abstracts, we then preliminarily selected 24 articles for full-text analysis. Following this, based on the inclusion and exclusion criteria, we further narrowed down the selection to 8 articles for quality evaluation. The process and results of the literature screening are depicted in Fig. 1.

### Quality assessment

Table 2 summarizes the quality assessment results of the eight identified studies. The majority of these studies described the phenomenon of interest, the typical participants, and the methods of data collection and analysis. Only two studies explicitly described the cultural or theoretical background of the researchers. Four studies addressed the researchers’ influence on the research process, such as through quality control measures, but failed to discuss the impact of the research on the researchers themselves. Only two studies did not explicitly state ethical approval or list the ethical approval number. Ultimately, two studies were assigned a grade A, six received a grade B, and all eight studies were included in the analysis.

### Characteristics of included studies

Eight studies were included after article screening and quality assessment. The studies were conducted in 6 countries; Canada (*n* = 2), Turkey (*n* = 2), New Zealand, Iceland, Ghana, and China. The methodologies used in the studies were 3 phenomenological studies [22, 27, 28], 1 ethnography study [29], 2 mixed studies [15, 30], and 2 qualitative studies with unspecified methodologies [31, 32]. A total of 122 multiparous pregnant women were included in the studies and the main method of data collection was interviews, both semi-structured and narrative; one study used observational methods. Data collection was postpartum in seven studies, and only one was collected antenatally [29]. Table 3 provides a detailed description of the included studies.

**Table 2:** Quality assessment of included studies in accordance

Study	Q1	Q2	Q3	Q4	Q5	Q6	Q7	Q8	Q9	Q10	Quality appraisal
Tahiru [15]	Y	Y	Y	N	Y	U	Y	Y	N	Y	B
Yang [22]	Y	Y	Y	Y	Y	N	U	Y	Y	Y	B
Kocabey [27]	Y	Y	Y	Y	Y	Y	Y	Y	Y	Y	A
Cinar [28]	Y	Y	Y	Y	Y	U	Y	Y	Y	Y	B
McKenzie [29]	U	Y	Y	N	Y	U	U	Y	Y	Y	B
Jonsdottir [30]	Y	Y	Y	Y	Y	Y	Y	Y	Y	Y	A
Leonard [31]	U	Y	Y	N	Y	N	N	Y	N	Y	B
McGovern [32]	U	Y	Y	Y	Y	U	N	Y	N	Y	B

Y = Yes; N = No; U = Unclear.

Q1 = Congruity between the stated philosophical perspective and the research methodology.

Q2 = Congruity between the research methodology and the research question or objectives.

Q3 = Congruity between the research methodology and the methods used to collect data.

Q4 = Congruity between the research methodology and the representation and analysis of data.

Q5 = Congruity between the research methodology and the interpretation of results.

Q6 = A statement locating the researcher culturally or theoretically.

Q7 = Influence of the researcher on the research, and vice-versa.

Q8 = Adequate representation of participants and their voices.

Q9 = Ethical approval by an appropriate body.

Q10 = Conclusions flowing from analysis, or interpretation, of the data.

**Table 3:** Key features and characteristics of 8 included studies

Study	Year	Country	Design (Data collection method)	Participants/Data collection	Phenomenon of interest	Main findings (Themes)
Tahiru [15]	2020	Ghana	Mixed method (in-depth interviews)	30 twin mothers/prenatal	To assess the prevalence of exclusive breast-feeding among twins and associated factors.	3 themes: reasons for choosing a daily breastfeeding frequency; cultural factors; exclusive breastfeeding for twins, and breastfeeding information provided in health education classes.
Yang [22]	2015	China	Phenomenology (semi-structured interviews)	24 twin mothers/prenatal	To understand the true experiences and needs of twin mothers breastfeeding	4 themes: lack of confidence in breastfeeding; fatigue in daily life; separation of mother and baby affects breastfeeding rates; hope for continued care and guidance after discharge.
Kocabey [27]	2022	Turkey	Phenomenology (semi-structured interviews)	13 twin mothers/prenatal	To determine the needs and underline the breastfeeding experiences of mothers with multiple babies.	3 themes: the meaning of being a mother of multiple babies and breastfeeding them; challenging life experiences during breastfeeding; the factors that affect motivation.
Cinar [28]	2013	Turkey	Phenomenology (narrative interview)	10 twin mothers/prenatal	To explore the needs and difficulties of mothers who had multiple babies.	6 themes: the willingness of mothers to breastfeed and continue; management of breastfeeding, use of pacifier; daily life, instructions of healthcare personnel; advice from practice of experienced mothers.
McKenzie [29]	2006	Canada	Ethnography (semi-structured initial interviews)	19 twin mothers/postpartum	Exploring and analyzing the information needs of twin women feeding their infants	4 themes: information seeking, breast feeding and good mothering; interpretative repertoires and the authority of baby-feeding information sources; helpful and unhelpful information; barriers to information seeking.
Jonsdottir [30]	2022	Iceland	Mixed method (individual semi-structured interviews)	14 twin mothers/prenatal	To explore the breastfeeding experiences of late preterm twin mothers	2 themes: the first month – a complex and strenuous phase where task‑oriented feeding regimes were followed; months two to four and navigation through feeding: finding your own path.
Leonard [31]	2002	Canada	Unspecific research methodology (observation)	9 mothers of triplets/prenatal	To promote hospital and community support for breastfeeding of infants with multiple births	7 themes: attitudes, breastfeeding resources, and goals; understanding maternal postpartum health status; individualizing rooming-in; initiating lactogenesis and breastfeeding; achieving sufficiency of breast milk production; coordinating breastfeeding care; hospital discharge of mothers and infants.
McGovern [32]	2014	New Zealand	Unspecific research Methodology (semi-structured interviews)	3 twin mothers/prenatal	Understand why mothers stop breastfeeding twins, and what can be done to support breastfeeding.	Three themes: mothers’ experience and knowledge; the importance of support; the challenges of breastfeeding twins.

### The breastfeeding experiences of mothers of multiples

After repeated reading, analysis, and comparison by the researchers, a total of 67 quotes were extracted, similar quotes were classified and combined into 11 categories, and further integrated into 4 integrated results.

#### Synthesized finding 1: The drive for breastfeeding

Breastfeeding is widely regarded as the best way to feed a newborn, providing numerous benefits for both the physical and psychological development of the infant and the mother [33, 34]. Breastfeeding is conventionally regarded as integral to ‘good mother‘ [29]. Several mothers of multiple births in the study noted that breastfeeding provided them with a sense of wellbeing and fulfillment, fostering intimate connection and enhancing their sense of maternal identity [27]. “I think breastfeeding provides such a bond. Breastfeeding reinforces bonding.” [27] Moreover, some mothers described breastfeeding as a ‘miracle’ and viewed it as a privileged experience [27]. “I believe that breastfeeding is a sacred thing. It is something really miraculous. As soon as the baby is born, s/he is looking for it, s/he finds it and sucks it.” [27] Consequently, these mothers often purchased breastfeeding equipment early on, illustrating their anticipation of breastfeeding [28]. “I bought a twin breastfeeding pillow and tried simultaneous breastfeeding but I couldn’t succeed.the babies were too small.” [28] The significance that multiple birth mothers attribute to breastfeeding helps sustain their determination to breastfeed despite challenges. “I think being a mother of twins is really a great privilege. It is also very difficult! This means we are very strong. what a blessing!” [27].

#### Synthesized finding 2: The challenges of breastfeeding multiple births

The second theme of the study examines the complex array of challenges encountered by multiparous women in the process of breastfeeding. These challenges encompass a range of physical and psychological obstacles, conflicts arising from balancing work responsibilities with breastfeeding commitments, and concerns related to the care of the newborn. These challenges affect the duration of breastfeeding and may even lead mothers to decide to stop breastfeeding.

#### Physical burden

The feeding duration for multiple births is significantly longer than that for single births. Prolonged feeding and caring for multiple births impose substantial physical strain, including symptoms such as lower back pain, insomnia, fatigue, and breast and nipple pain [22, 27, 28, 30]. “I was in bed, and I had a catheter and could not walk or talk. I was sort of out of it for about two days after birth, and I could not see my children ” [30]“My nipples were cracked” [28]. In addition, mothers of multiple births with older children have to juggle older children and multiple newborns, making breastfeeding multiple births overwhelming. “After the cases where I broke in tears, I got up immediately and told myself that I have to be strong, I have to do it, I have to accomplish it. In the end, they’re my children.” [27].

Thus, for women with multiple births, the physical exhaustion following a caesarean section conflicts with the physical and energetic demands of breastfeeding multiples, leading to significant maternal stress. Additionally, the frequency of suckling sessions for multiple breastfeeding infants increases substantially compared to single births, elevating the risk of breast and nipple pain.

#### Psychological burden

Some interviewees also talked about their psychological pressure, such as overwhelm, anxiety, guilt, disappointment and depression [22, 27, 28, 30]. Some mothers feel confused and overwhelmed about breastfeeding multiple babies after the birth of newborns or when hospitalized newborns return home because of the complexity of breastfeeding and the physical challenge of feeding multiple babies [30]. Even some women gave up breastfeeding: “Although I know breastfeeding is good, when I really face two children, I feel that my mind is empty, and I always feel afraid that they will be hungry, so I just feed milk powder. ” [22]. However, parturients with low milk supply regretted that they could not extablish exclusive breastfeeding [30]. However, mothers with experience in breastfeeding consider successful breastfeeding experience to be very important for subsequent breastfeeding of twins [32].

Therefore, some women find breastfeeding burdensome and want to return to their normal routine and family relationships. “Then, I thought, ‘This is starting to be a burden’ it cannot overshadow the time spent with the family and other things I want to do. ” [30].

There are also some mothers who consider their own mother’s identity, so they can remind themselves to be strong in the face of poor physical and psychological conditions [27, 31]. “After the cases where I broke in tears, I got up immediately and told myself that I have to be strong, I have to do it, I have to accomplish it. In the end, they’re my children. ” [27].

In most studies, multiple birth mothers have negative emotional experiences of breastfeeding, with some women viewing breastfeeding as a “burden” and longing to return to normal life, while others choose to continue breastfeeding and view it as a “problem” to be overcome.

#### The burden of immature feeding patterns

The incidence of premature birth in multiple births is higher than that in single births, and newborns who can be discharged with the mother are mostly late preterm or early term infants, with weak sucking ability and prone to sleepiness. The mother believes that breastfeeding is time-consuming and difficult, requiring more patience. “You know they sucked a little and then just went to sleep; they were so tired.” [30]; “The oldest is less than 4 catties and will not breastfeed after discharge” [22]. As a result of the infants’ immature feeding pattern, breastfeeding becomes complicated, leading to a loss of confidence in breastfeeding among multiple birth mothers.

Multiple births are fed more frequently than single births, and unfortunately the immature feeding patterns of newborns make them more difficult to feed, and the challenge of “making it harder” adds to the negative feelings of multiple births.

#### The burden of work-breastfeeding conflict

Similar to single-child mothers, the mothers’ jobs are also important factors affecting the choice of multiple-child mothers to continue breastfeeding. Balancing work and breastfeeding is a challenging issue for mothers. “Because I have to go to work, I really can’t help but give up.” [22]. “My work is so involving and I need to make money hence my inability to let them suckle much.” [15].

Following a series of physical and psychological challenges, the end of maternity leave, and conflicts between work and breastfeeding can make work an ‘opportunity’ or ‘turning point’ for weaning.

#### Synthesized finding 3: Stage management and individualised adaptation of breastfeeding

The third theme was stage management and individualised adaptation of breastfeeding, and this study demonstrated that mothers with multiples have different coping strategies in the early and mature stages of the breastfeeding process.

#### Coping strategies in the early stages of feeding

In the early stages of breastfeeding, the management of breastfeeding in mothers of multiples is characterized by “prudent” feeding and efforts to increase lactation [30]. Mothers of multiples with separated babies or preterm infants also frequently use equipment to establish lactation or actively go to the hospital to deliver milk [22, 30]. “Prudent feeding” involves strict adherence to standardized intervals between feeds, precise measurements of milk volume, monitoring weight gain and loss, tracking baby excretions, and scheduling nappy changes. “We rented a scale that we placed on this table and weighed them. We wrote down how much they weighed and, of course, calculated how much they needed in addition to breastfeeding. After four days, we felt this was insane.” [30]. Mothers of multiples may often manage their milk supply tightly in the early stages [27, 29, 30], using a breastpump to empty the breasts after breastfeeding and managing inadequate milk supply by changing breasts or dividing the milk equally [28–30]. “After every feeding for many weeks after birth, I used the breast pump to get more milk.” [30] “There was more milk on the right side.the fatty baby (3,000 g) was sucking that side; the other one was 2,650 g. I thought that was unjust.thus, I interchanged the breasts.” [28] Mother-infant separation or immature feeding patterns increase the likelihood of using breast pumps, hand milking, or nipple shields to stimulate milk production.

Multiple birth mothers face particular challenges in the early stages of feeding, and their commitment to breastfeeding their babies is further reflected in their thorough feeding programmes and use of equipment to maintain lactation.

#### Adaptation shifts during feeding maturation

After mastering the early and complex breastfeeding management and the maturation of infant feeding patterns, some mothers became skilled in breastfeeding techniques and developed individualized feeding routines based on each infant’s needs and feeding bond [30]. However, some mothers experienced fatigue with breastfeeding, gradually stopped pumping, and began accepting formula feeds after breastfeeding. This eventually led to the cessation of breastfeeding due to decreased milk production [30]. “During the second month, I quit pumping. I just had enough and found it easier to just breast- and bottle feed. Pumping interrupted my sleep at night and just everything. We had three weeks where it was going fine, but suddenly, there was no milk.” [30].

Most mothers can find an individual way to breastfeed during the breastfeeding maturation period, although some may experience a decrease in milk supply and eventually stop breastfeeding.

#### Synthesized finding 4: The experience of supports

Multiple birth mothers receive support for breastfeeding from family, friends, socia, medical, and information, but these supports don’t exactly promote breastfeeding for multiple births. The following section discusses women’s experiences of receiving this support.

#### Family support

As the number of babies increases, the time spent on breastfeeding and other infant care tasks also increases. Therefore, women with twins or triplets often express the need for more family support to help them complete breastfeeding successfully [27, 30, 32]. Spouses or family members assisting in preparing formula, taking care of other infants, and providing emotional support during feeding allow mothers to spend more time on extended breastfeeding and find it meaningful. However, as the husband returns to work, this support diminishes, and the demanding task of caring for multiple infants leaves the mother fatigued and unable to continue breastfeeding [30]. “We could not have done this without my parents. My mother sat by their [the twins] side while they slept so we could take a nap” [30]. Differences in childcare beliefs between mothers and their family members can create discord within the family unit [27]. “. It would be better if the helper were closer to my mind set. I got angry because my mother-in-law was very involved. I asked her to stay out of it.because I want to make my own decisions” [27].

Some mothers of multiples also seek support and help from friends and experienced “elders” in the local area [15, 30]. “My friend who lives close by sometimes dropped by during lunchtime. She, of course, got coffee, but she also fed them [solids] and gave them a bottle [laughs]. It somehow turned into this: If someone comes, then he or she is handed the bottle.” [30].

Mothers of multiple births require direct or indirect breastfeeding support from their social networks, including family and friends, to ensure they have the time and energy to breastfeed.

#### Social support

A New Zealand study mentions that the government provides 240 h of non-means-tested family support services over 12 months for families with children under five and then twins [32]. And these family support services are domestic services such as cleaning, cooking and laundry [35]. Additionally, twin mothers can benefit from the establishment of relevant social service groups or peer support groups, which can offer emotional and other forms of breastfeeding support. Mothers of multiples believe that the situation of feeding multiple children is different from that of single-child mothers, so they are eager to make friends with other multiple-child mothers to obtain peer support [29]. “And I’ve had some of them say, ‘Look, I have a girlfriend who had twins, this is her phone number’.” [29].

Social support from government organizations, social service groups, and peer support groups is essential for helping mothers of multiple births maintain breastfeeding.

#### Medical support

Professional medical support as a part of social support is very important for the success and duration of breastfeeding for mothers of multiple children. All participants in the study believed that receiving prenatal training from medical professionals was helpful for achieving breastfeeding. Additionally, they required sufficient medical support following discharge [28]. Mothers who are separated from their babies often face difficulties and doubts in breastfeeding due to lack of experience with multiple infants and not receiving professional breastfeeding guidance. As a result, they require more continuous medical support [31]. “Two babies were born prematurely and transferred to pediatrics. After returning home, the baby did not eat (breast milk) at all, but the eldest learned it as soon as possible, so only one was breastfeeding.” [31]. In two studies, respondents had a contradictory psychological state of worrying about disturbing medical staff but also desiring continuous medical services [28, 31]. “They just answered me as I questioned.they didn’t give information on their own.” [28]. However, the unprofessional feeding support and unencouraging feeding attitude of medical staff can also indirectly lead to multiple women not being able to achieve exclusive breastfeeding [27].

In terms of medical support for breastfeeding, multiple birth mothers expect professional antenatal education, continuity of medical support, proactive medical care and positive attitudes towards breastfeeding.

#### Information support

Most studies have mentioned the need for information support during various stages of breastfeeding, as well as the problems encountered in seeking information support [15, 22, 29–31]. This study will be presented from four perspectives: the source, characteristics, content, and dissemination form of information.

Regarding the source of information, compared to feeding guidance from community workers, mothers trust the feeding knowledge provided by hospital medical staff more. “After returning home, I had engorged breasts, so I hired a lactation consultant, but the effect was not good. When community workers visited, I was more willing to believe in your guidance.” [22]. However, some mothers did not receive information about breastfeeding multiple children, so they turned to the internet, friends or “elders” around them for help [30].

In terms of the characteristics of information, some mothers of multiple children believe that their feeding experiences are different from those of single-child mothers and that each mother and baby is unique. However, the feeding information provided in prenatal classes and by medical staff is not targeted at multiple births, and medical staff do not provide personalized guidance for each mother. “I said, ‘I know that probably everyone, everyone else was expecting one but’ I said, ‘can you cover twins a little bit for me? ’” [29].

In terms of the content of information, mothers of multiple children usually hope to get a detailed explanation of a range of topics, including the benefits of breastfeeding, the possibility of breastfeeding multiple children, how to breastfeed two infants at the same time, how to balance rest with breastfeeding, how family members can support mothers of multiple children in breastfeeding, and how to increase milk production [29, 30]. “I’m worried about one getting up, and then you just nurse them and feed them and then you fall asleep and then the other one’s getting up, and then it’s like you’d be so tired.” [29].

One study mentioned that mothers of multiple children prefer to receive information through written promotional brochures as a medium. “Instructions should start during pregnancy. an illustrated booklet would be very good” [28].

Mothers of multiple births who sought information about breastfeeding rated their experiences with various information sources, personalisation, content, and media support. They expressed a preference for personalised information from health professionals regarding breastfeeding multiples. Additionally, they found an easily accessible illustrated booklet to be an effective medium for information support.

## Discussion

This study comprehensively describes the real experiences and diverse needs of women with multiple births concerning breastfeeding throughout the perinatal period and at all stages of the future implementation of feeding, including four aspects: willingness to breastfeed, multiple challenges in the implementation of breastfeeding multiple births, stage management and individualised adaptation of breastfeeding, and the evaluation of support received.

Breastfeeding intention reflects the attitudes and beliefs of multiparous mothers toward breastfeeding. A positive intention facilitates their active pursuit of breastfeeding information and the establishment of related behaviors, thereby enhancing both the duration and quality of breastfeeding. A study has shown that antenatal intention to breastfeed is a predictor of six-month breastfeeding rates in pregnancies with twins [14]. Our study revealed that while the majority of multiparous mothers demonstrated a strong inclination to breastfeed, a subset remained ambivalent. The findings suggest that professional prenatal education significantly boosts breastfeeding intentions [36]. Accordingly, medical staff should not only encourage a positive breastfeeding disposition but also respect individual preferences, providing tailored information to those without an initial intention to breastfeed [37]. The Queensland Organization Breastfeeding Guidelines 2023 [37] recommend that healthcare providers assess barriers to breastfeeding at delivery, offer detailed guidance on infant feeding practices at subsequent deliveries, and collaborate with mothers to formulate and document comprehensive breastfeeding plans. Additionally, multiparous mothers are consulted about their previous breastfeeding experiences; if negative, strategies to address these issues are discussed [37].

Women with multiple births, as evidenced in five studies, frequently report fatigue and strain during breastfeeding [22, 27, 28, 30, 31]. Postpartum, woman’s recovery will be affected by physical and psychological factors, such as a weakened postpartum state, frequent breastfeeding and sleep deprivation. Common complaints among women with multiple births include lower back pain, fatigue, sleep deprivation, nipple soreness, anxiety, and increased incidences of crying, leading to adverse attitudes toward breastfeeding. Furthermore, research indicates that multiple births correlate with diminished postnatal mental health compared to single births [5], and negative emotional states can further decrease lactation and milk supply [38]. To mitigate these challenges, medical and mental health professionals should prioritize multiparous mothers for follow-up visits. These should focus on educating them about the stress-relieving benefits of oxytocin released during breastfeeding, acknowledging and addressing their physical and psychological hardships, and providing strategies to prevent and manage physical discomfort. This approach aims to bolster their confidence and promote successful breastfeeding [38, 39].

Compared with full-term infants, infants from multiple pregnancies often experience developmental challenges such as inadequate sucking, lethargy, and poor feeding tolerance, which complicate breastfeeding [40]. These challenges can diminish the self-confidence of multiparous women and exacerbate anxiety and other negative emotions [22]. Furthermore, current antenatal breastfeeding promotion primarily addresses term and singleton births, lacking detailed guidance for multiple births. A meta-analysis demonstrated that breastfeeding interventions grounded in the Theory of Planned Behavior or the Theory of Breastfeeding Self-Efficacy significantly enhance breastfeeding rates and durations [41]. Medical professionals should therefore implement evidence based grounded in psychological theory programs, offering tailored breastfeeding guidance that addresses the specific behaviors of the newborn and the physiological condition of the mother. This guidance should include techniques for assessing effective suckling, measuring actual breastfeeding amounts, scheduling feedings, and incorporating breastfeeding enhancements such as breastmilk fortifiers. For complex cases, prompt referral to breastfeeding specialists or certified lactation consultants is crucial.

Research analyzing mothers with multiple births who breastfed beyond 12 months indicates that those with partner and family support experienced longer breastfeeding durations compared to their unsupported counterparts [42]. Thus, robust family and social support plays a critical role in promoting successful breastfeeding among women with multiple births. Emotional, parenting, and household support from partners or family members significantly enhance the ability of mothers with multiple births to allocate more time to breastfeeding. Such support also promotes adequate rest and relaxation, which facilitates oxytocin secretion and reduces the risk of insufficient milk production. The Breastfeeding Co-Parenting Theoretical Framework [43] underscores the importance of partner or co-parent involvement in breastfeeding success and provides guidance on effective support strategies. The framework comprises five components, providing support ranging from the emotional to the practical dimensions. In the emotional dimension, examples include co-parents’ willingness to participate in breastfeeding, offering encouragement and praise to mothers of multiples, and addressing challenges faced during childcare and feeding in a positive manner. In the practical dimension, support includes providing breastfeeding-related information, necessary feeding equipment, and ensuring a fair distribution of daily childcare and household chores. This equitable distribution allows mothers of multiples to have the time and energy needed for breastfeeding. Consequently, healthcare professionals should discuss parenting styles with mothers of multiples and their families during the antenatal and postnatal periods, actively coordinating breastfeeding and emotional support based on this framework. This approach highlights how critical co-parenting support is to the longevity and success of breastfeeding. Currently, healthcare workers lack adequate knowledge about breastfeeding multiples, revealing a significant gap between medical practice and optimal multiple breastfeeding support. This gap hinders healthcare workers from effectively delivering personalized breastfeeding assistance to mothers of multiples. Addressing this gap requires enhancing the training and education of medical personnel to improve their competence and professionalism in breastfeeding support.

The New Zealand Government’s non-means-tested domestic support for families with multiple births can help families with multiple births to focus more on breastfeeding and parenting, and increase breastfeeding rates [32]. Currently, non-profit organizations such as The Multiple Births Foundation, Twins Trust, and Twins & Multiple Births Association offer some free social services to these mothers. Healthcare professionals should provide specific information and counseling about these resources when assisting families with multiple, thereby enhancing their access to social support.

Excellent medical support for breastfeeding is multifaceted. Healthcare workers must possess a comprehensive understanding of multiple birth breastfeeding to effectively assist mothers in selecting and initiating appropriate infant feeding practices, as well as in addressing subsequent issues [44]. It is essential for healthcare professionals to exhibit commendable personal qualities, respect the feeding choices of women with multiples, affirm their decisions confidently, and offer guidance on suitable methods [37]. Moreover, clinical staff require advanced communication skills to engage with mothers using acceptable and effective methods [37]. Additionally, it is crucial for healthcare workers to develop and implement feasible, evidence-based breastfeeding intervention programs specifically tailored for multiple births [45]. Such programs should leverage distance learning [46], peer education [47], and visual aids like graphic booklets or videos [48] to enhance breastfeeding rates among mothers of multiples.

In the UK, health visitors offer support advice and guidance to families with children under five. However, families with multiple births often find that the generalized support from health visitors fails to address the complexities of breastfeeding multiples [49]. The Elizabeth Bryan Centre for Multiple Births advocates for providing families with health visitors who have specialized training in multiple births [50]. The health visitor service, which facilitates the transition from hospital to home, is crucial. In countries lacking such services, hospitals should establish multidisciplinary follow-up teams to ensure continuity of care for families with multiple and support breastfeeding mothers. Therefore, both health visitors and medical follow-up team members need to pursue systematic education on multiple breastfeeding, such as studying specialized guidelines [51], to offer scientifically sound, authoritative, and customized support for managing breastfeeding in multiple births and assisting mothers through all stages of breastfeeding.

### Strengths and limitations

This study constitutes the first systematic review of qualitative research synthesis concerning breastfeeding among women with multiple births, offering significant clinical relevance. We meticulously recorded the outcomes of the original studies, and through the aggregation and analysis of codes, were able to effectively mitigate content bias. In addition, all members of this study had undergone systematic evidence-based training, which enabled them to professionally review and assess the quality of the literature. Moreover, during the process of the study, the research members kept in touch with each other to discuss and analyse the results, which effectively improved the credibility of the whole study. The literature included in this study involved countries with both high and low rates of breastfeeding, so the findings may have a wider application. We have included suggestions for improving care in Table 4.

However, the study also has several limitations. The results of the meta-synthesis were influenced by the limited number of qualitative studies on the subject of breastfeeding in multiple births—specifically, eight studies—of which six were rated Grade B and only two were Grade A for high quality. In addition, this study did not include qualitative articles with the theme of real-life experiences of parenting in women with multiple births, which also affected the comprehensiveness of the results to a certain extent, considering that descriptions of breastfeeding might be mentioned in their studies.

**Table 4:** Recommendations for improved care

Category	Specific Recommendations
Breastfeeding Intention	1. Respect the feeding intentions of mothers with multiples. 2. Encourage positive breastfeeding intentions as much as possible.
Physical and Psychological Stress	1. Helping mothers to rationalise physical and psychological stress 2. Provide appropriate preventive and curative measures for physical complaints 3. Provide psychological support or referral to psychological clinics to increase confidence in breastfeeding.
Immature Feeding Patterns of Infants	1. Provide personalised feeding advice based on the newborn’s feeding behaviour and the mother’s condition. 2. Refer mothers with complex breastfeeding problems to breastfeeding specialists or International Board Certified Lactation Consultants (IBCLCs).
Family Support	Actively coordinate emotional and practical support for mothers of multiples within a shared parenting framework.
Social Support	1. Provide domestic services to families with multiples without economic assessment. 2. Provide information and consultation on non-profit organizations specializing in breastfeeding for twins/multiples.
Medical Support	1. Train health professionals in breastfeeding for multiples and related skills. 2. Ensure personal qualities and effective communication that respect mothers’ feeding choices. 3. Develop feasible, evidence-based breastfeeding intervention plans, providing information and medical support using effective interventions. 4. Train health visitors in breastfeeding for multiples or set up multidisciplinary follow-up teams to provide ongoing breastfeeding support after discharge.

## Conclusion

This study employs a meta-synthesis approach to examine the experiences and needs of women breastfeeding twins or higher-order multiples, aiming to offer targeted recommendations. The findings suggest that these women generally report negative experiences and encounter numerous challenges. Consequently, it is advised that hospitals establish multidisciplinary follow-up teams involving obstetrics, neonatology, and community care for these women. These teams should develop interventions based on theoretical frameworks to support breastfeeding in multiple-birth scenarios. Furthermore, families with multiple births should be treated as educational units to ensure continuous, personalized care that includes promoting feeding information before delivery, providing guidance after delivery, and monitoring breastfeeding post-discharge.

Future relevant interventional trials and multicentre cohort studies on breastfeeding multiples are needed to assess the contribution of different interventions to multiple breastfeeding rates and to inform subsequent guideline development by specialist in breastfeeding for multiple births.

### Electronic supplementary material

Below is the link to the electronic supplementary material.

Supplementary Material 1

## Data Availability

No datasets were generated or analysed during the current study.

## References

[1] Tang W, Zou L. Trends and characteristics of multiple births in Baoan Shenzhen: a retrospective study over a decade. Front Public Health. 2022;10:1025867. 36582383 10.3389/fpubh.2022.1025867 PMC9793989

[2] Quitadamo PA, Comegna L, Palumbo G, Copetti M, Lurdo P, Zambianco F et al. Feeding Twins with Human Milk and Factors Associated with its duration: a qualitative and quantitative study in Southern Italy. Nutrients. 2021; 13(9). 10.3390/nu13093099 PMC8464927 34578976

[3] Martin JA, Hamilton BE, Osterman M, Driscoll AK, Births. Final data for 2019. Natl Vital Stat Rep. 2021;70(2):1–51. 33814033

[4] Multifetal Gestations. Twin, Triplet, and higher-Order Multifetal pregnancies: ACOG Practice Bulletin, Number 231. Obstet Gynecol. 2021;137(6):e145–62. 34011891 10.1097/AOG.0000000000004397

[5] Fenwick N, Chorley A. Twin and higher-order pregnancy – patient Voice. In: Khalil L, Lopriore, editor. Twin and higher-order pregnancies. Cham: Springer International Publishing; 2021. pp. 425–35.

[6] Merter OS, Altay N. Effect of feeding fresh or frozen breast milk on the gut microbiota of premature infants: a prospective observational study. Biol Res Nurs. 2024;26(1):78–90. 37626020 10.1177/10998004231191728

[7] Section OB, Eidelman AI, Schanler RJ, Johnston M, Landers S, Noble L, et al. Breastfeeding and the use of human milk. Pediatrics. 2012;129(3):e827–41. 22371471 10.1542/peds.2011-3552

[8] Yokoyama Y, Wada S, Sugimoto M, Katayama M, Saito M, Sono J. Breastfeeding rates among singletons, twins and triplets in Japan: a population-based study. Twin Res Hum Genet. 2006;9(2):298–302. 16611502 10.1375/twin.9.2.298

[9] Ooki S. The effect of an increase in the rate of multiple births on low-birth-weight and preterm deliveries during 1975–2008. J Epidemiol. 2010;20(6):480–8. 20827033 10.2188/jea.JE20100022 PMC3900826

[10] Anjarwati N, Waluyanti FT, Rachmawati IN. Exclusive breastfeeding for Twin babies and its influencing factors: a study in East Java, Indonesia. Compr Child Adolesc Nurs. 2019;42(sup1):261–6. 31192718 10.1080/24694193.2019.1594458

[11] Shim JI, Kang SJ. Impact of Breastfeeding Knowledge, attitude, and barriers on breastfeeding practice among Twin mothers. Korean J Women Healt. 2017;23 2:89–98. 10.4069/kjwhn.2017.23.2.89 37684888

[12] Ostlund A, Nordstrom M, Dykes F, Flacking R. Breastfeeding in preterm and term twins–maternal factors associated with early cessation: a population-based study. J Hum Lact. 2010;26(3):235–41. 20139377 10.1177/0890334409359627

[13] Wang S, Li M, Xiang X, Guo X, Peng C, Wang D, et al. Analysis on the current situation of twin breastfeeding and its influencing factors. Med. 2023;102(38):e35161. 10.1097/MD.0000000000035161 PMC10519451 37746974

[14] Odei JA. Factors Associated with Exclusive Breastfeeding of Ghanaian Twins. Ghana: University of Ghana; 2013.

[15] Tahiru R, Agbozo F, Garti H, Abubakari A. Exclusive Breastfeeding and Associated Factors among Mothers with Twins in the Tamale Metropolis. Int J Pediatr. 2020; 2020:5605437. 10.1155/2020/5605437 PMC6996674 32099551

[16] World Health Organization. Breastfeeding. https://www.who.int/health-topics/breastfeeding#tab=tab_3

[17] Ceylan S, Şahin S. Comparison of breastfeeding self-efficacy and breastfeeding success of obese and normal-weight mothers in the early period. Afr Health Sci. 2020;20:2022–31. 34394268 10.4314/ahs.v20i4.60 PMC8351850

[18] Cinar N, Kose D, Alvur M, Dogu O. Mothers’ attitudes toward Feeding Twin babies in the First Six months of life: a sample from Sakarya, Turkey. Iran J Pediatr. 2016;26(5):e5413. 28203331 10.5812/ijp.5413 PMC5297257

[19] Bartick M, Reinhold A. The burden of suboptimal breastfeeding in the United States: a pediatric cost analysis. Pediatrics. 2010;125(5):e1048–56. 20368314 10.1542/peds.2009-1616

[20] Mikami F, Francisco R, Rodrigues A, Hernandez WR, Zugaib M, de Lourdes BM. Breastfeeding twins: factors related to Weaning. J Hum Lact. 2018;34(4):749–59. 29660295 10.1177/0890334418767382

[21] Huaijie Y, Tao W, Xiaoyun L, Wenfei Z, Xiaoyu Y. [The current situation investigation and analysis about breastfeeding of twins]. Hebei Med. 2019;41(3):463–5.

[22] Huaijie Y, Qiong D, Wenhua L, Aihua C, Lu H, Liqiong Z, et al. [A qualitative study of the breastfeeding experiences and needs of twin mothers]. Chin Nurs Manag. 2015;15(09):1051–4.

[23] Tong A, Flemming K, Mcinnes E, Oliver S, Craig J. Enhancing transparency in reporting the synthesis of qualitative research: ENTREQ. BMC Med Res Methodol. 2012;12:181. 23185978 10.1186/1471-2288-12-181 PMC3552766

[24] Lockwood C, Munn Z, Porritt K. Qualitative research synthesis: methodological guidance for systematic reviewers utilizing meta-aggregation. JBI Evid Implement. 2015; 13(3). 10.1097/XEB.0000000000000062 26262565

[25] Thomas J, Harden A. Methods for the thematic synthesis of qualitative research in systematic reviews. BMC Med Res Methodol. 2008;8:45. 18616818 10.1186/1471-2288-8-45 PMC2478656

[26] Foyston Z, Higgins L, Smith DM, Wittkowski A. Parents’ experiences of life after medicalised conception: a thematic meta-synthesis of the qualitative literature. Bmc Pregnancy Child. 2023;23(1):520. 10.1186/s12884-023-05727-x PMC10351127 37460955

[27] Kocabey Z, Karakoç A. Breastfeeding experience of mothers with multiple babies: a phenomenological study. Clin Exp Health Sci. 2022;12(1):18–25. 10.33808/clinexphealthsci.753034

[28] Cinar ND, Alvur TM, Kose D, Nemut T. Breastfeeding twins: a qualitative study. J Health Popul Nutr. 2013;31(4):504–9. 24592592 10.3329/jhpn.v31i4.20049 PMC3905645

[29] Mckenzie PJ. The seeking of baby-feeding information by Canadian women pregnant with twins. Midwifery. 2006;22(3):218–27. 16697092 10.1016/j.midw.2005.03.006

[30] Jonsdottir RB, Flacking R, Jonsdottir H. Breastfeeding initiation, duration, and experiences of mothers of late preterm twins: a mixed-methods study. Int Breastfeed J. 2022;17(1):68. 36076279 10.1186/s13006-022-00507-3 PMC9461222

[31] Leonard LG. Breastfeeding higher order multiples: enhancing support during the postpartum hospitalization period. J Hum Lact: Official J Int Lactation Consultant Association. 2002;18(4):386–92. 10.1177/089033402237914 12449057

[32] Mcgovern T. The challenges of breastfeeding twins. Kai Tiaki Nurs New Z. 2014;20(11):26–44. 25668866

[33] Li R, Ware J, Chen A, Nelson JM, Kmet JM, Parks SE et al. Breastfeeding and post-perinatal infant deaths in the United States, a national prospective cohort analysis. Lancet Reg Health Am. 2022; 5. 10.1016/j.lana.2021.100094 PMC9335131 35911656

[34] Yuen M, Hall OJ, Masters GA, Nephew BC, Carr C, Leung K, et al. The effects of breastfeeding on maternal Mental Health: a systematic review. J Womens Health (Larchmt). 2022;31(6):787–807. 35442804 10.1089/jwh.2021.0504

[35] Government Assistance. Multiples NZ. https://multiples.org.nz/government-assistance/

[36] Menekse D, Çinar N. The Effect of Breastfeeding Education Provided to pregnant women who expect twins on their breastfeeding intention. Online Türk Sağlık Bilimleri Dergisi. 2022;7:4. 10.26453/otjhs.1097111

[37] Queensland Clinical Guidelines. Establishing breastfeeding. Queensland Health. 2021. http://www.health.qld.gov.au/qcg

[38] Nagel EM, Howland MA, Pando C, Stang J, Mason SM, Fields DA, et al. Maternal psychological distress and lactation and breastfeeding outcomes: a narrative review. Clin Ther. 2022;44(2):215–27. 34937662 10.1016/j.clinthera.2021.11.007 PMC8960332

[39] Henshaw EJ. Breastfeeding and Postpartum Depression: A Review of relationships and potential mechanisms. Curr Psychiatry Rep. 2023;25(12):803–8. 37906349 10.1007/s11920-023-01471-3

[40] Russell S, Russell N. Breastfeeding Twins and multiples. In: Khalil L, Lopriore, editor. Twin and higher-order pregnancies. Cham: Springer International Publishing; 2021. pp. 355–62.

[41] Chipojola R, Chiu HY, Huda MH, Lin YM, Kuo SY. Effectiveness of theory-based educational interventions on breastfeeding self-efficacy and exclusive breastfeeding: a systematic review and meta-analysis. Int J Nurs Stud. 2020;109:103675. 32585447 10.1016/j.ijnurstu.2020.103675

[42] Monvillers S, Tchaconas A, Li R, Adesman A, Keim SA. Characteristics of and sources of support for women who Breastfed multiples for more than 12 months. Breastfeed Med. 2020;15(4):213–23. 32058794 10.1089/bfm.2019.0237

[43] Abbass-Dick J, Dennis CL. Breast-feeding Coparenting Framework: a New Framework to improve breast-feeding duration and exclusivity. Fam Community Health. 2017;40(1):28–31. 27870751 10.1097/FCH.0000000000000137

[44] Wallenborn JT, Lu J, Perera RA, Wheeler DC, Masho SW. The impact of the Professional qualifications of the prenatal care provider on Breastfeeding Duration. Breastfeed Med. 2018;13(2):106–11. 29236524 10.1089/bfm.2017.0133

[45] Whitford HM, Wallis SK, Dowswell T, West HM, Renfrew MJ. Breastfeeding education and support for women with twins or higher order multiples. Cochrane Database Syst Rev. 2017;2(2):CD012003. 28244065 10.1002/14651858.CD012003.pub2 PMC6464508

[46] Moukarzel S, Caduff A, Rehm M, Del FM, Perez-Escamilla R, Daly AJ. Breastfeeding Communication Strategies, challenges and opportunities in the Twitter-Verse: perspectives of influencers and Social Network Analysis. Int J Environ Res Public Health. 2021; 18(12). 10.3390/ijerph18126181 PMC8230232 34201000

[47] Rodriguez-Gallego I, Leon-Larios F, Corrales-Gutierrez I, Gonzalez-Sanz JD. Impact and Effectiveness of Group Strategies for Supporting Breastfeeding after birth: a systematic review. Int J Environ Res Public Health. 2021; 18(5). 10.3390/ijerph18052550 PMC7967547 33806469

[48] Novianty N, Syarif S, Ahmad M. Influence of breast milk education media on increasing knowledge about breast milk: literature review. Gac Sanit. 2021;35(Suppl 2):S268–70. 34929828 10.1016/j.gaceta.2021.10.031

[49] Turville N, Alamad L, Denton J, Cook R, Harvey M. Supporting multiple birth families: perceptions and experiences of health visitors. Public Health Nurs. 2022;39(1):229–37. 34761411 10.1111/phn.13008 PMC9299212

[50] Turville N, Alamad L, Denton J, Harvey M. Supporting multiple birth families; establishing an evidence base to inform Health Visitor Practice. Twin Res Hum Genet. 2021;24(6):392.

[51] Foundation MB. Guidance for health professionals on feeding twins, triplets and higher order multiples. Multiple Births Foundation; 2015.

